# Application of Lignin-Containing Cellulose Nanofibers and Cottonseed Protein Isolate for Improved Performance of Paper

**DOI:** 10.3390/polym14112154

**Published:** 2022-05-25

**Authors:** Jacobs H. Jordan, Michael W. Easson, Huai N. Cheng, Brian D. Condon

**Affiliations:** The Southern Regional Research Center, Agricultural Research Service, USDA, 1100 Allen Toussaint Blvd., New Orleans, LA 70124, USA; jacobs.jordan@usda.gov (J.H.J.); hn.cheng@usda.gov (H.N.C.); brian.condon@usda.gov (B.D.C.)

**Keywords:** cottonseed protein, cellulose nanofibers, lignin, paper, dry strength

## Abstract

There is current interest in replacing petroleum-based additives in consumer paper products with abundantly available, renewable and sustainable biopolymers such as lignin-containing cellulose nanofibers (LCNFs) and cottonseed protein. This research characterized the performance of cottonseed protein isolate with/without LCNFs to increase the dry strength of filter paper. The application of 10% protein solution with 2% LCNFs as an additive improved the elongation at break, tensile strength and modulus of treated paper products compared to the improved performance of cottonseed protein alone. Improvements in tensile modulus and tensile strength were greatest for samples containing larger amounts of lignin and a greater degree of polymerization than for those with less lignin from the same biomass sources.

## 1. Introduction

Paper is used for a variety of consumer applications and products including printing, writing, tissues, towels, newsprint, packaging and paperboard. The physical property demands of these different applications and products often require the use of additives that enhance their performance properties [1,2,3]. Typically, these additives are used to improve paper dry and wet strength performance. Different additive types include cationic starch and acrylamide for dry strength [4] and formaldehyde resins, poly(amino-amide)-epichlorohydrin resins and polyacrylamide polymers for wet strength [5]. However, due to acute toxicity and persistent environmental concerns associated with these compounds, non-toxic, biodegradable and environmentally friendly paper additives are becoming increasingly preferred [3].

Several environmentally friendly additives such as soy protein [6,7,8,9,10] have been previously reported for use in paper products as binders and strength agents. Others include gelatin, zein, hydroxyproline-rich glycoprotein [11] and cottonseed protein (CSP) [12]. Cottonseed protein isolate is currently used in animal feed [13,14] and for the formulation of biobased products [15,16,17]. Other applications of cottonseed protein include films, coatings and adhesives [16]. The performance of cottonseed protein in adhesive applications was improved by the addition of anionic polysaccharides [18], amino acids [19], carboxylic acids [12], phosphorus-containing compounds [20] or nanocellulose [21].

Another environmentally friendly additive, nanocellulose can be processed into cellulose nanocrystals (CNCs) or cellulose nanofibers (CNFs) by mechanical or chemical treatments of purified cellulose fibers [22,23,24]. Improved wet and dry performance was observed of laboratory prepared paper sheets when derivatized carboxymethylated CNFs and poly(aminoamide)-epichlorohydrin were used [25], or when treated with periodate oxidized CNCs [26]. Additionally, CNFs obtained from cotton gin motes (CGM) improved the dry strength of paper products when applied at a 2% additive level with 10% CSP, producing improvements in tensile strength and a modulus of 15–20% [27].

In addition to paper products, CNFs have found applications in biomedical devices [28,29], tissue engineering [30,31] and as a reinforcement material in polymers, films, gels, foams and other composites [32,33,34,35,36]. Unlike petroleum-based fibers, CNFs are nontoxic, do not persist for extended periods of time in the environment and are completely biodegradable [37,38]. However, the production of CNFs is often temporally and financially costly, and it would be beneficial to obtain CNFs from less highly purified cellulose fibers.

With this in mind we recently reported the production of lignin-containing cellulose nanofibers (LCNFs) from cotton gin motes (CGM) and cotton gin trash (CGT) containing varying amounts of lignin content (3–18%), which could meet this low-cost need as a paper reinforcement additive by reducing costly chemical processing and bleaching [39]. The aim of this study is to determine the efficacy of using LCNFs from two biomass sources—CGM and CGT—as an additive with CSP to enhance the mechanical properties of treated paper samples compared to CNFs that have been chemically processed, containing a lower percentage of lignin and hemicellulose.

## 2. Materials and Methods

### 2.1. Materials

Cotton gin motes (CGM) and cotton gin trash (CGT) were supplied by the USDA research facility in Stoneville, MI, USA. Cottonseed protein isolate was prepared from the defatted seeds of glandless cottonseed by the base solubilization and acid precipitation procedure previously reported [19,40]. All other reagents and supplies were purchased from Millipore Sigma (St. Louis, MO, USA) or VWR (Radnor, PA, USA) unless otherwise stated and used without further purification. For all protein formulations, ultra-pure water with a minimum resistance of 18.2 MΩ was used (0.055 µS/cm at 25 °C).

### 2.2. Isolation of Lignocellulose Biomass

Lignocellulose biomass was isolated from raw CGM and CGT using the procedures reported previously [39,41]. Briefly, biomass samples were prepared with differing lignin contents by separate mechanical and chemical processing of CGM and CGT [39,41]. CGM and CGT were first milled to <20 mesh with a Wiley mill (E3300, Eberbach Corp., Belleville, MI, USA) and the obtained powders were treated for 2 h at 60 °C with a 4% (*w*/*w*) sodium hydroxide (NaOH) solution at a fiber to liquor ratio of 1:20 (*w*/*v*). The fibers were then washed with deionized water until a neutral eluant (pH ≈ 7) was achieved. The solids were dried to obtain biomass samples from CGM containing approximately 9% lignin and 88% cellulose and hemicellulose or samples from CGT containing 64% cellulose/hemicellulose and 18% lignin. The lignocellulose products were reserved for the production of unbleached LCNFs with greater lignin content (GM-LCNF with 9% lignin produced from CGM and GT-LCNF with 18% lignin produced from CGT) [39].

To further reduce lignin content, lignocellulose biomass was exhaustively bleached at 75 °C for 2 h with an acidified sodium chlorite solution (1.0% acetic acid (*v*/*v*), 0.50% NaClO_2_ (*w*/*v*)), which was repeated twice for CGM and three times for CGT until the fibers were white. Cellulose was recovered after thorough washing with deionized water. Each solid portion was then dried to a constant mass at 70 °C to produce cellulosic solids from CGM with 95% cellulose/hemicellulose and 3% lignin or cellulosic solids from CGT with 87% cellulose/hemicellulose and 6% lignin, which was used to prepare, respectively, GM-CNFs with 3% lignin and GT-CNFs with 6% lignin [39].

### 2.3. Preparation of Cellulose Nanofibers

Suspensions of CNFs and LCNFs were prepared using a combination of wet-disk milling and microfluidization techniques as described previously [39,41]. Each solid portion was suspended in deionized water at approximately 1% (*w*/*w*) using an Ultra-Turrax^®^ (T25, IKA Works, Inc., Wilmington, NC, USA) mechanical homogenizer. The slurry was passed through a Supermasscolloider MKCA6-2 (Masuko Sangyo Co., Ltd., Saitama, Japan) with a disk clearance of 4 µm and a rotational speed of 12,000 rpm for ten successive passes, which defibrillates the fibers by high-shear forces [33]. The obtained nanocellulose slurry was then subjected to high-shearing forces using a high-pressure homogenizer (Microfluidizer M-110EH, Microfluidics Corp., Newton, MA, USA). The suspension was pumped through one 200 µm ceramic and one 87 µm diamond Z-shaped interaction chamber for a total of five passes with an operating pressure of 2097 MPa. This provided CNFs with a lignin content of 18% (GT-LCNF), 9% (GM-LCNF), 6% (GT-CNF) and 3% (GM-CNF) in the solid fractions, respectively, where the notation for LCNFs is used to denote CNFs with a greater lignin content (9% or 18%). Specifically, GM-CNF and GT-CNF were obtained from bleached cellulosic fibers obtained from CGM or CGT, while GM-LCNF and GT-LCNF were obtained from unbleached cellulosic fibers. After each nanocellulose suspension was collected, the concentration was adjusted to 1.0 wt% prior to use.

### 2.4. Preparation of Treated Paper Samples

#### 2.4.1. Preparation of Cottonseed Protein and Nanocellulose Formulations

For the preparation of the CSP formulations, CSP was suspended in deionized water and the CSP suspension was stirred for 60 min. To the stirred suspension was added 1.0 wt% CNF (or LCNF) slurry at a CSP:CNF (or LCNF) ratio of 50:1. The sample was then diluted to produce a final CSP concentration of 10% (*w*/*w*). For the CSP-only formulation, only deionized water was added. Each mixture (CSP, CSP + CNF or CSP + LCNF) was then homogenized with a Silverson L5-MA high shear Laboratory mixer. Prior to applications, the pH of each sample was adjusted to pH 10.5 with small aliquots of 2.0 M NaOH solution.

#### 2.4.2. Treatment of Paper Samples

For each paper sample, Whatman #1 filter paper manufactured from cotton linters was used with a manufacturer specified thickness of 180 µm. Paper sheets were cut into 1″ × 6″ (2.54 cm × 15.24 cm) strips, immersed in deionized water to remove water-soluble contaminants and allowed to dry under ambient conditions.

Strips were then evenly coated with a mixture containing CSP (10% *w*/*w*, pH 10.5) or CSP with CNFs (or LCNFs). Each formulation was applied to seven paper strips using a soft brush, and the treated strips of paper were then dried under ambient conditions. The dried strips were heat-pressed (0.25 MPa) for 10 min at 120 °C using a heated benchtop press (Model 3856, Carver Inc., Wabash, IN, USA). The dry weight of the paper strips was measured prior to and after the application of the protein/nanocellulose formulations. The paper thickness was measured before and after application of the formulations with a digital precision thickness gauge (FT3, Hanatek Instruments, East Sussex, UK). Prepared paper strips were subsequently characterized with thermogravimetric analysis (TGA) optical microscopy and scanning electron microscopy (SEM).

### 2.5. Thermogravimetric Analysis (TGA)

Thermogravimetric (TG) and differential thermogravimetric (DTG) analyses were performed under a nitrogen atmosphere using a TGA Q500 thermal gravimetric analyzer (TA Instruments, New Castle, DE, USA). The nitrogen flow into the furnace was maintained at a rate of 90 mL·min^–1^. A small square was cut from the paper samples weighing approximately 4–6 mg, which was placed into a platinum crucible. The samples were heated from approximately 30 °C to a target temperature of 600 °C with a heating rate of 10 °C min^−1^. The obtained thermogravimetric traces for TG and DTG were analyzed with Universal Analysis 2000 software (TA Instruments). Each sample analysis was performed in triplicate. The curves were averaged, and the resulting curves were plotted with OriginPro 2018b software (OriginLab Corp., Northampton, MA, USA).

### 2.6. Microscopic Analysis of Paper

#### 2.6.1. Scanning Electron Microscopy (SEM)

The surface morphology of paper samples was obtained using a field emission scanning electron microscope (FE SEM, Hitachi 4800, Tokyo, Japan). Each sample was sputter-coated for 15 min with a vacuum sputter coater to apply a thin layer of carbon. The data were collected at an acceleration voltage of 3 keV and a beam current of 0.5 nA.

#### 2.6.2. Optical Microscopy

Images were collected using a Hirox KH-8700 3D Digital Microscope with Auto XY Stage Controller (Hirox-USA, Inc., Hackensack, NJ, USA). Tiling and 3D images were constructed using twenty-five layered 3.2–4.0 µm stacked subsections.

### 2.7. Analysis of Paper Samples

For the analysis of paper dry strength, a method was adapted from ASTM D 828-97 [12,42]. Briefly, paper dry strength measurements were performed with a Zwick stress tester (Zwick GmbH & Co., Ulm, Germany). During the analysis the crosshead speed was 1 mm·min^–1^, and data were collected for the tensile modulus, the tensile strength and the maximum elongation at break. Seven paper strips were analyzed for each formulation, and analyses were repeated in (at least) triplicate. Differences in the paper’s mechanical properties were determined using an analysis of variance (ANOVA) and a Tukey’s means comparison test (α = 0.05).

## 3. Results and Discussion

### 3.1. Preparation of Formulations and Treatment of Paper Samples

**Figure 1:**
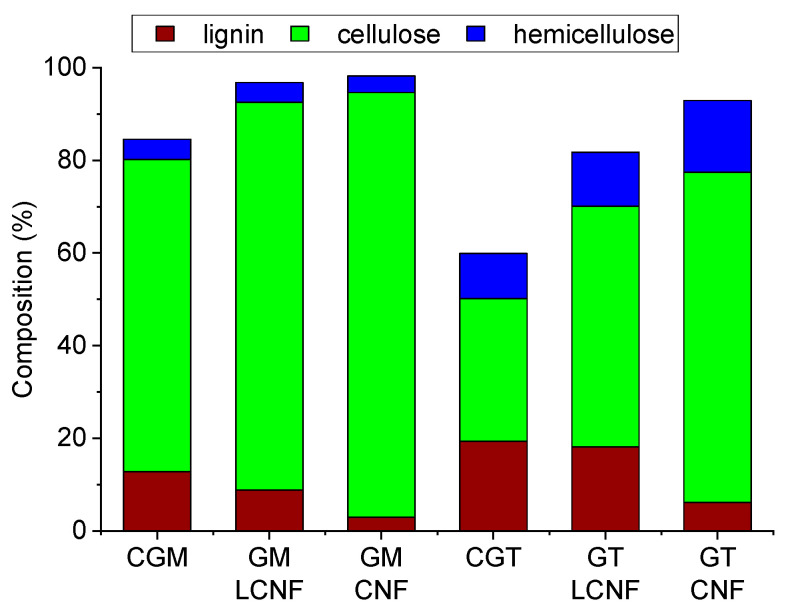
Composition (%) of cotton gin motes (CGM), cotton gin trash (CGT) and cellulose nanofibers (CNFs).

Cellulose nanofibers with different lignin contents were prepared from CGM and CGT as described previously [39]. To achieve different content of lignin in the products for application as performance additives, CGM or CGT were selected at different stages of processing. Figure 1 shows the composition of the as-received CGM and CGT [41,43], as well as the composition of the materials used for preparation of CNFs and LCNFs [39].

For the application of additive to paper samples, the concentrations of CSP (10%) and nanocellulose (0.2%) were chosen consistent with prior results [27]. Notably, greater concentrations of nanocellulose were attempted; however, they were unsuccessful. This is due in part to the larger degree of polymerization of the unbleached LCNF samples and both CGM samples compared to the fully bleached samples derived from CGT (GT-CNF). Additionally, CGM samples (GM-CNF and GM-LCNF), with a longer degree of polymerization, more readily agglomerate in suspensions, further increasing viscosity [39]. Thus, the application of greater concentrations of nanocellulose additives above 0.2% with 10% CSP resulted in increased suspension viscosity such that a consistent application was not possible between different nanocellulose/CSP formulations.

**Figure 2:**
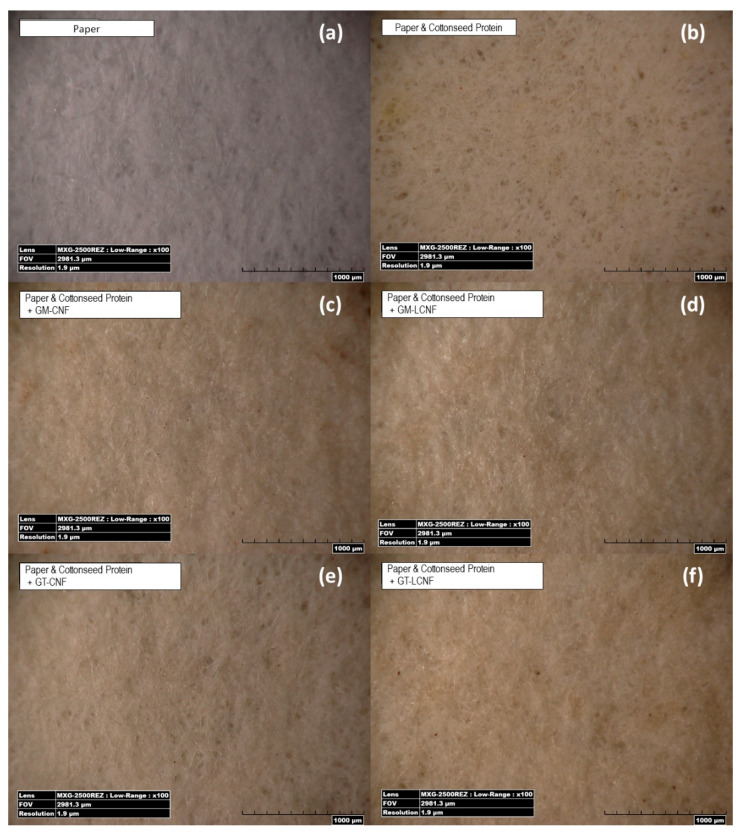
Optical microscopy images (100× magnification) of (**a**) control paper and paper treated with (**b**) cottonseed protein isolate (CSP); (**c**) CSP and GM-CNFs; (**d**) CSP and GM-LCNFs; (**e**) CSP and GT-CNFs; and (**f**) CSP and GT-LCNFs.

Control formulations of paper samples were treated with only a dilute NaOH solution, and each treated paper sample was prepared as described in the Materials and Methods section. The application of CSP and each CNF/LCNF formulation provided an even distribution of the protein and nanofibers across the paper surface, as shown by optical microscopy (Figure 2).

**Figure 3:**
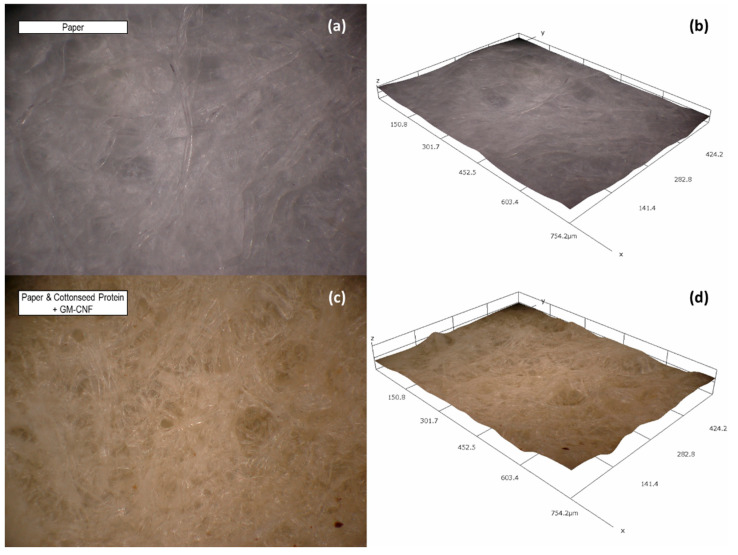
(**a**) 3D composite image (400×) of paper and (**b**) 3D-layered topographical map (400×); (**c**) 3D composite image (400×) of paper treated with cottonseed protein and cotton gin mote cellulose nanofibers and (**d**) 3D layered topographical map (400×).

The further examination of the paper surface with optical microscopy (Figure 3a) revealed a randomly distributed and uniform surface morphology with only minor variation in the surface structure (Figure 3b). When formulations of CSP were applied to paper, 3D composite images confirmed a uniform deposition of the CSP and CNFs (Figure 3c) evenly distributed throughout the paper, with no change in the paper structure or morphology without disrupting the surface structure. This indicates that the formulations are dispersed throughout the samples rather than applied only as a surface coating.

### 3.2. Characterization of Paper Samples

**Figure 4:**
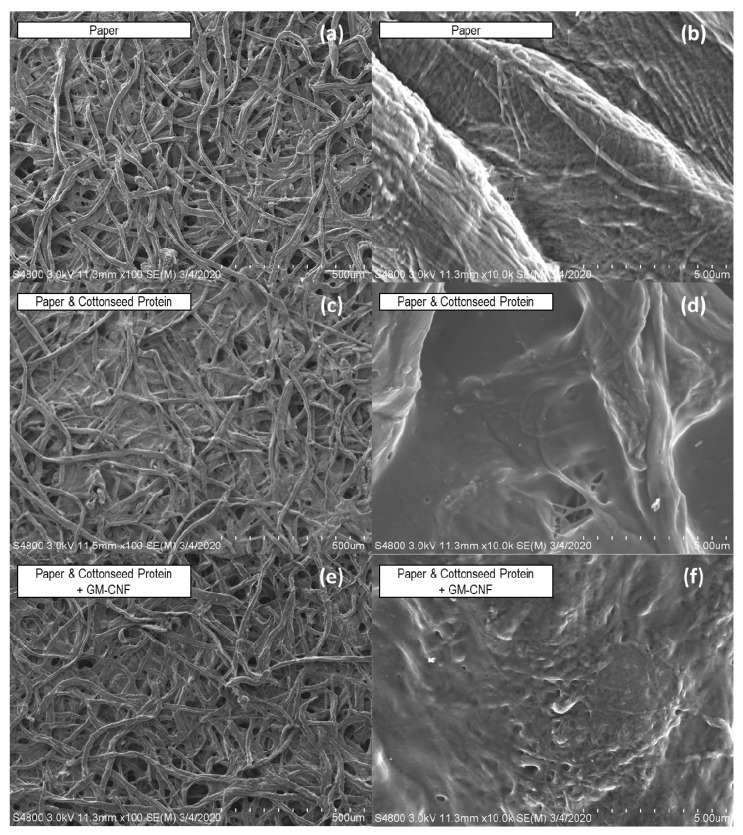
FESEM micrographs at 100× (**a**,**c**,**e**) and 1000× (**b**,**d**,**f**) of paper (**a**,**b**) and paper treated with 10 wt% CSP solution (**c**,**d**) and 0.2 wt% GM-CNF and 10 wt% CSP (**e**,**f**).

To further examine the application of CSP and CNF formulations onto paper substrates, field emission scanning electron microscopy (FE-SEM) was used to obtain images of the fibers’ morphology (Figure 4). Images were acquired at the same magnification (100×) as those obtained using optical microscopy and indicate a loose network of randomly distributed fibers for the filter paper and treated paper samples. A representative sample of the paper treated only with CSP (Figure 4c,d) and with CSP with GM-CNF (Figure 4e,f) is shown. A close examination of the FE-SEM photomicrographs indicates structure with numerous void spaces and the fine details associated with the microfibrillar structure of the cotton fibers at greater magnifications (Figure 4b) [27,44]. The treatment of the paper with CSP indicates adherence of the protein to the fiber’s structure, which fills the small voids; as such, the protein can be seen to effectively coat cotton fibers and interfibrillar spaces, which results in a smoother appearance for the cotton fibers (Figure 4d,f). Although not shown, the results were similar for other CSP formulations.

The application of the solution for the paper samples produced no significant change in thickness and weight; the total weight change was 0.3%, which is less than the error in the measurement, and the thickness was measured at 175.1 ± 9.5 µm, which agrees with the manufacturer’s specified thickness of 180 µm. Paper samples treated with different formulations of CSP and CNFs (or LCNFs) indicated no change in sample thickness compared to treatment with only CSP (Table 1). The treated paper thickness varied between 202 and 216 µm with a coefficient of variation of 9–11%. For sample dry weight add-ons (weight pick-up), samples treated with each CSP formulation gained on average 30% weight pick-up, and differences between formulations were not considered significant. Therefore, changes ultimately expressed in the performance of the paper samples can be attributed to differences in the nanocellulose/CSP formulations and not attributed to variations in paper thickness, weight add-on, or any variation in the amount or application of the CSP formulations or final dimensions of the treated paper samples.

**Figure 5:**
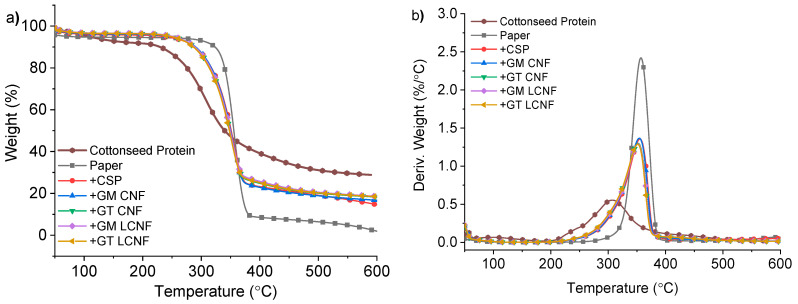
(**a**) TGA and (**b**) DTG data for CSP (brown), paper (black), paper + CSP (red), paper + CSP+GM-CNF (blue), paper + CSP + GT-CNF (green), paper + CSP + GM-LCNF (violet) and paper + CSP + GT-LCNF (gold); DTG—differential thermogravimetry; TGA—thermogravimetric analysis.

Thermogravimetric analysis was performed on paper samples treated with the different CSP and LCNF formulations, and the results are displayed in Figure 5. Moreover, the TGA thermogram for plain paper and CSP is also shown in Figure 5. The TGA thermogram for plain paper is consistent with that obtained for pure cellulose [27,41,45,46] indicated by a sharp thermal decomposition around 340 °C and very little char residue (<2%) remaining in the paper samples. In contrast, the samples of solid cottonseed protein isolate exhibited a broad thermal degradation over the range of approximately 260–380 °C (T_onset_ = 262.5 °C) and produced considerable (>30%) char residue, which is consistent with prior reports (see Table 2 for details) [12,27,44].

The application of CSP and CSP formulations with CNFs (or LCNFs) lowered the initial T_onset_ compared to plain paper alone; however, in nearly all instances, there was no significant difference observed between the samples treated with CSP and CSP-CNF (or LCNF) formulations with the TGA and DTG traces essentially overlapping in both thermograms. In the DTG thermogram for all treated paper samples (Figure 5b), the DTG trace for the CSP peak merged with that of the main cellulose decomposition instead of the formation of a separate peak in the DTG thermogram; this indicates a degree of interaction between the protein and the paper substrate [44]. The average T_onset_ for all treated paper samples was between 313 °C and 319 °C. However, it can be noted that for some samples treated with LCNF with a greater lignin content, the T_onset_ was marginally suppressed, and the corresponding T_max_ obtained from the DTG trace was lowered. This can possibly be attributed to the samples containing larger percentages of lignin and hemicellulose. A similar trend was observed previously for the analysis of LCNF samples containing gradient lignin content [39]. Overall, the average char residue was unaffected by the percentage of lignin present in the CNF/LCNF samples or to the different percentages of lignin present in the CNF/LCNF samples when applied with CSP and produced an average char residue of ~20% in all instances for treated paper strips.

### 3.3. Paper Analysis

**Figure 6:**
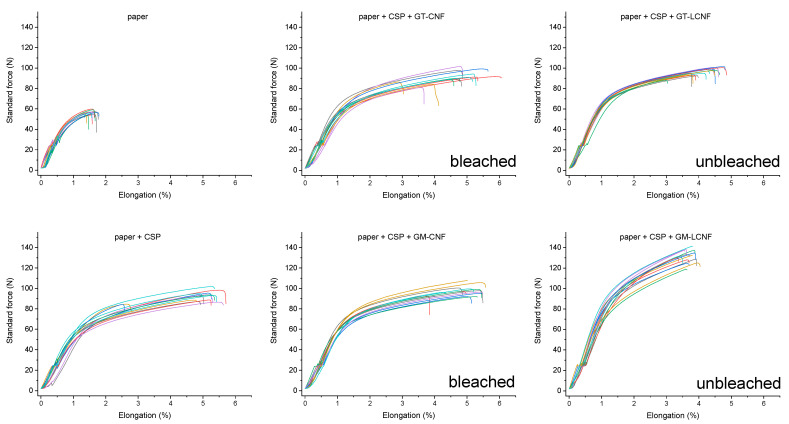
Stress–strain curves for various filter paper samples for control paper group and after treatment with formulations of cottonseed protein isolate (CSP) and cellulose nanofibers (CNFs) or lignin-containing cellulose nanofibers (LCNFs) from cotton gin motes (GM) or gin trash (GT).

**Figure 7:**
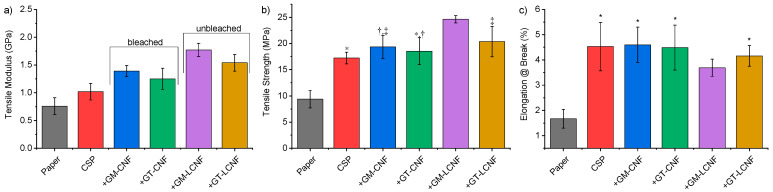
Results of mechanical testing of paper treated with cottonseed protein and cellulose nanofiber dispersions: (**a**) tensile modulus; (**b**) tensile strength; (**c**) elongation at break. * Note: Each data point represents seven strips tested in triplicate (21 total tests per entry). Data bars with the same symbol (*, †, or ‡) indicate the treatments are not significantly different at *p* < 0.05.

Plain paper and treated paper strips were analyzed for their tensile modulus, elongation at break and tensile strength using a Zwick stress tester, and the resulting stress-strain curves are shown in Figure 6 (The full data file is available in the Supplementary Information). The analysis of results of dry strength tensile testing of the paper strips are reported in Figure 7, indicating the tensile modulus, tensile strength and maximum elongation at break. Paper strips treated with the control formulations of only a dilute NaOH solution produced a tensile modulus of 0.76 ± 0.15 GPa, a tensile strength 9.39 ± 1.67 MPa and an elongation at break of (1.67% ± 0.35%), which is consistent with an earlier report [27]. The application of CSP to the paper strips produced an improved tensile modulus (1.02 ± 0.15 GPa), tensile strength (17.23 ± 1.12 MPa) and elongation at break (4.53% ± 0.96%) compared to plain paper.

It can be noted that, in all cases, the application of CSP and CNF/LNCF formulations similarly resulted in an improvement of the paper mechanical properties (modulus and tensile strength) compared to the application of CSP alone, while the elongation at break remained essentially constant, approximately 4.45% ± 0.74%. The formulations of CSP with 2% nanofiber additive from bleached GM-CNF and GT-CNF increased the tensile modulus by 36% (1.39 ± 0.10 GPa) and 25% (1.25 ± 0.19 GPa). In contrast, the unbleached source of nanofibers had a greater effect, with GT-LCNF producing a 51% greater modulus (1.54 ± 0.15 GPa) than the application of CSP. The greatest effect was observed for the samples of GM-LCNF, which had the greatest tensile modulus (1.77 ± 0.12 GPa) and tensile strength (24.63 ± 0.69 MPa), although this sample was the only sample to exhibit a reduced elongation at break (3.69 ± 0.34%) compared to the treatment with CSP isolates. This represents an improvement of >130% compared to plain paper alone and is 76% greater than that observed for only the application of CSP in terms of the tensile modulus. Tensile strength is 167% greater. In terms of tensile strength, the other samples exhibited a tensile strength of 18–21 MPa and were improved compared to the application of CSP alone. Specifically, the tensile strength of samples prepared with GM-CNF and GT-CNF provided comparable values with 19.34 ± 2.20 MPa and 18.54 ± 2.56 MPa, respectively, while GT-LCNF produced further improved tensile strengths (20.38 ± 2.90 MPa).

Each unbleached nanofiber sample containing a greater percentage of lignin from CGM or CGT, when used as an additive, outperformed the samples of CNFs prepared from the fully bleached source material. This is advantageous since the greater lignin content LCNFs were obtained from unbleached cellulose sources and, thus, represent an attractive energy and cost-saving alternative to highly purified CNFs when used as a strength modifier. A possible explanation of this result is that the oxidative bleaching process may have affected CNF’s performance properties, since oxidative bleaching reduces the overall degree of polymerization and length of the cellulose chains; a larger degree of polymerization has been attributed to increased modulus and yield stress in cellulose nanopaper [47]. The observed trend, however, was nonmonotonic; the degree of polymerization for CNFs/LCNFs is GT-CNF < GT-LCNF < GM-CNF < GM-LCNF, while the mechanical properties increase in the order of GT-CNF ≈ GM-CNF < GT-LCNF < GM-LCNF [39]. This nonmonotonic trend can be partly explained by the greater percentage hemicellulose (Figure 1) in the samples derived from CGT (GT-CNF and GT-LCNF), so the observed effect is reduced as the heterogenous and amorphous materials impart less of a strengthening effect than do pure cellulose fibers, since these materials have reduced sample crystallinity [39,43], which may affect macroscopic properties including strength and toughness.

An analysis of wet paper strength was similarly performed using a method adapted from ASTM D 829-97. The treated paper strips were prepared as described, and then they were immersed in distilled water for 1 h at 23 °C. Immediately after saturation with water, the strips were tested for strength. However, the samples treated with CSP and each CSP-CNF/LCNF formulation exhibited no differences in their mechanical properties (data not shown). This, however, is consistent with observations from earlier reports [12,27] where CSP was used as a strength modifier for paper products. In previously reported results, no enhancement was observed for paper wet-strength testing when combined with aspartic, adipic and citric acid, and in a later report, wet-strength testing indicated no significant difference between other cellulose nanomaterial formulations and those obtained with CSP.

## 4. Conclusions

This research has shown that, in conjunction with cottonseed protein, cellulose nanofibers obtained from fully bleached cellulose and those possessing a greater lignin content from unbleached cellulose sources can be used as a strength modifier for paper products. The application of cottonseed protein isolate with nanocellulose dispersions was shown to improve paper dry strength performance, improving the tensile modulus and tensile strength of treated paper by >130% and 167%, respectively. No significant changes were observed in paper wet strength performance compared to the application of cottonseed protein alone. This work suggests that lignin-containing cellulose nanofibers are viable low-cost alternatives as a supplement to cottonseed protein for improvement of paper strength.

## Figures and Tables

**Table 1:** Physical properties of paper samples treated with cottonseed protein and nanocellulose formulations.

Sample	Thickness (µm) *	Weight Pick-Up (%) *
Paper	175.06 ± 9.53	0.3 ± 1.7
Paper and 10% CSP ^1^	202.12 ± 22.09 ^a^	28.2 ± 2.5 ^a^
+0.2% GM-CNF ^1,2^	204.84 ± 23.98 ^a^	32.0 ± 6.1 ^a^
+0.2% GT-CNF ^2^	207.40 ± 22.25 ^a^	28.9 ± 4.3 ^a^
+0.2% GM-LCNF ^3^	215.88 ± 23.43 ^a^	31.5 ± 4.3 ^a^
+0.2% GT-LCNF ^3^	201.12 ± 17.54 ^a^	30.0 ± 4.3 ^a^

* Data with the same superscript letter. ^a^ indicate that treatments are not significantly different at *p* < 0.05. ^1^ This number is consistent with a prior report [27]. ^2^ CNF samples were obtained from bleached cellulosic fibers. ^3^ LCNF samples were obtained from unbleached cellulosic fibers.

**Table 2:** Thermal properties of cottonseed protein isolate and paper samples treated with cottonseed protein and cellulose nanofiber formulations.

Sample	T_onset_ *	T_max_ *	Char *
CSP	262.5 ± 0.5	307.2 ± 0.3	31.5 ± 0.4
Paper	337.5 ± 0.6	357.1 ± 0.6	1.9 ± 0.1
Paper and 10% CSP ^1^	318.2 ± 0.6 ^a^	354.6 ± 1.0 ^a^	19.8 ± 0.7 ^a^
+0.2% GM-CNF ^1,2^	317.5 ± 1.1 ^a^	353.9 ± 1.0 ^a^	19.9 ± 1.0 ^a^
+0.2% GT-CNF ^2^	315.9 ± 1.2 ^a,b^	353.3 ± 0.8 ^a,b^	19.5 ± 0.8 ^a^
+0.2% GM-LCNF ^3^	315.1 ± 2.4 ^a,b^	352.5 ± 1.6 ^a,b^	19.9 ± 0.8 ^a^
+0.2% GT-LCNF ^3^	313.7 ± 0.4 ^b^	351.5 ± 0.2 ^b^	20.2 ± 0.4 ^a^

* Data with the same superscript letter (^a^, or ^b^) within a column indicate the treatments are not significantly different at *p* < 0.05. ^1^ This number is consistent with a prior report [27]. ^2^ CNF samples were obtained from bleached cellulosic fibers. ^3^ LCNF samples were obtained from unbleached cellulosic fibers.

## Data Availability

Tensile testing stress-strain curves may be found in the Supplementary Information file: data_file.xlsx.

## Supplementary Materials

The following supporting information can be downloaded at: https://www.mdpi.com/article/10.3390/polym14112154/s1, Data File: data_file.xlsx.
